# Insights from ecological niche modeling on the taxonomic distinction and niche differentiation between the black-spotted and red-spotted tokay geckoes (*Gekko gecko*)

**DOI:** 10.1002/ece3.1183

**Published:** 2014-08-18

**Authors:** Yueyun Zhang, Chongtao Chen, Li Li, Chengjian Zhao, Weicai Chen, Yong Huang

**Affiliations:** 1 Guangxi Botanical Garden of Medicinal PlantsNanning, 530023, Guangxi, China; 2 Nanning ZooNanning, 530003, Guangxi, China; 3 Natural History Museum of GuangxiNanning, 530012, Guangxi, China

**Keywords:** Climate, ecological speciation, *Gekko gecko*, *Gekko reevesii*, MaxEnt, niche divergence, taxonomy

## Abstract

The black-spotted tokay and the red-spotted tokay are morphologically distinct and have largely allopatric distributions. The black-spotted tokay is characterized by a small body size and dark skin with sundry spots, while the red-spotted tokay has a relatively large body size and red spots. Based on morphological, karyotypic, genetic, and distribution differences, recent studies suggested their species status; however, their classifications remain controversial, and additional data such as ecological niches are necessary to establish firm hypotheses regarding their taxonomic status. We reconstructed their ecological niches models using climatic and geographic data. We then performed niche similarity tests (niche identity and background tests) and point-based analyses to explore whether ecological differentiation has occurred, and whether such differences are sufficient to explain the maintenance of their separate segments of environmental ranges. We found that both niche models of the black- and the red-spotted tokay had a good fit and a robust performance, as indicated by the high area under the curve (AUC) values (“black” = 0.982, SD = ± 0.002, “red” = 0.966 ± 0.02). Significant ecological differentiation across the entire geographic range was found, indicating that the involvement of ecological differentiation is important for species differentiation. Divergence along the environmental axes is highly associated with climatic conditions, with isothermality being important for the “black” form, while temperature seasonality, precipitation of warmest quarter, and annual temperature range together being important for the “red” form. These factors are likely important factors in niche differentiation between the two forms, which result in morphological replacement. Overall, beside morphological and genetic differentiation information, our results contribute to additional insights into taxonomic distinction and niche differentiation between the black- and the red-spotted tokay.

## Introduction

It is well known that many species can be difficult to diagnose and delimit, especially when using single operational criterion such as morphological characters or DNA markers (de Queiroz 2007). Using multiple lines of evidence such as integrating morphological and molecular analyses can resolve many taxonomic uncertainties. In addition, recent proposals have called for combining ecological niche approaches, spatially explicit analyses of environmental data, and phylogenetics in cryptic species delimitation (Rissler and Apodaca 2007). If morphologically similar species are characterized by distinct genetic lineages that also occur under different ecological conditions, this strengthens support for their treatment as distinct species (Wielstra et al. 2012). More and more studies suggested that the addition of ecological niche modeling for taxa into phylogeographic surveys provides further insights into species taxonomy and distributions of organisms (*Aneides flavipunctatus*, Rissler and Apodaca 2007; *Phelsuma*, Raxworthy et al. 2007; *Phrynosoma*, Leaché et al., 2009; *Calotes versicolor*, Huang et al. 2013).

It has long been hypothesized and widely acknowledged that ecological processes can constrain species distributions and determine the geographic location of range limits (Darwin 1859; Orr and Smith 1998). Species range limits are separated by the species ecological niche (Sexton et al. 2009), which are often found to be associated with sharp or gradual spatial gradients in environmental factors (e.g., temperature, precipitation) and are defined by a set of, for example, habitat structure, climate, resources, and predators or competitors pairs (Holt and Keitt 2005). In such cases, closely related species may specifically adapt to environmental conditions with their respective preferred habitats (Khimoun et al. 2012). If so, divergent selection acts on populations due to the different environmental conditions and consequently may drive the evolution of reproductive isolation between populations occurring in allopatry or sympatry (Schluter 2001). Therefore, the general mechanism (i.e., ecological speciation) might promote phenotypic divergence and reproductive isolation. This may result in population pairs being more genetically divergent and differing in a greater number of adaptive phenotypic traits to local environmental conditions (Schluter 2001; Nosil and Sandoval 2008).

Ecological niche modeling (ENM) offers a powerful tool that uses ecological information extracted from species distributions and of ecological processes driving spatial patterns of biodiversity (Graham et al. 2004; Elith et al. 2006). These methods can predict the potential distribution of species by combining species occurrence data with environmental data (Elith et al. 2006). Such methods may provide insights into the most important environmental variables that maintain distributions, thus affecting the geographic limits of genetic lineages. Consequently, ENM methods enable us to test whether ecological niches are different or identical between species (Warren et al. 2008). Therefore, results from ecological niche modeling may elucidate the causal relationships between environmental variables, ecological divergence in phenotypic traits, and speciation (Rissler and Apodaca 2007).

**Figure 1:**
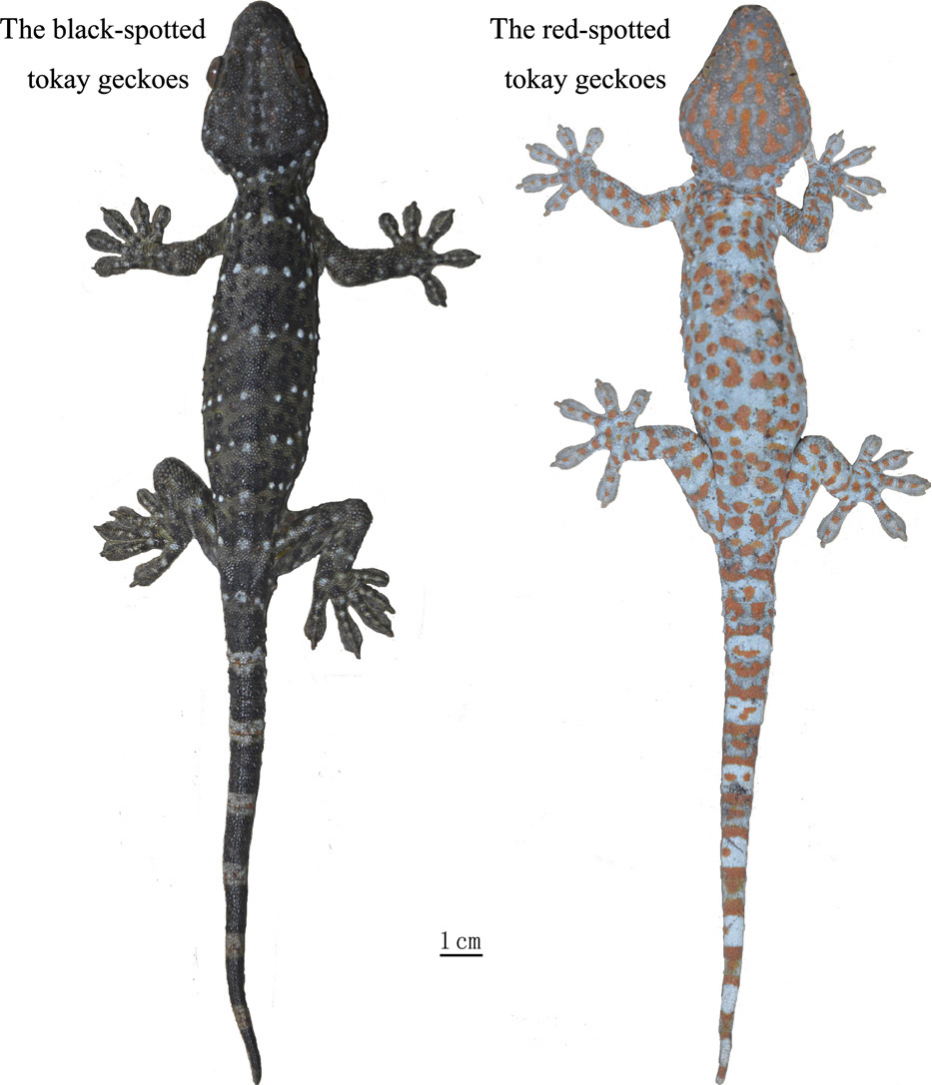
Body size and spots differences between the black- and the red-spotted tokay. The “black” form has a small body size and dark skin with sundry spots. In contrast, the “red” form has a larger body size and marked red spots.

The tokay gecko, *Gekko gecko* (Linnaeus 1758), is polytypic, including two subspecies, *G. g. gecko* and *G. g. azhari*. *Gekko gecko gecko* is widely distributed across southern China and most other countries in Southeast Asia. It has also been introduced into America, and several Caribbean islands as an invasive species. *Gekko gecko azhari* is restricted to Bangladesh. Two forms of *G. g. gecko*, the black- and the red-spotted tokay (termed “black” form and “red” form henceforth), have long been recognized because of their morphological differences (Zhang et al. 1997; Chan et al. 2006; Qin et al. 2007, 2009). The “black” form has a small body size and dark skin with sundry spots. In contrast, the “red” form has a larger body size and marked red spots (Fig.1). Morphological variability in the two morphs is inheritable and relatively stable (Zhang et al. 1997). Liu et al. (2000), Zhang et al. (2006), Qin et al. (2007, 2012) suggested that the two forms are genetically differentiated by phylogenetic analyses using mitochondrial DNA. Wang et al. (2012) suggested that the “red” lineage and one of the “black” lineages have probably differentiated into two subspecies; however, lineages within the “black” form were not monophyletic. Moreover, the “red” and the “black” forms also showed different karyotypes on the 15th chromosome (Qin et al. 2012). Using the available comprehensive morphological, karyotypic, genetic, and zoogeographical results, Rösler et al. (2011) suggested that the “black” form should be regarded as a separate species, *G. reevesii*. However, previous studies have not considered ecological niche differences, which could be useful to establish stable hypotheses of their taxonomic relationships.

Given that the classification of these two taxa remains controversial, here we (1) develop ecological niche models using georeferenced museum locality information and environmental data layers; (2) furthermore, examine whether ecological differentiation has occurred between the “black” and the “red” forms in order to provide additional insight into their taxonomic status; (3) gain a better understanding of how abiotic factors (e.g., temperature and precipitation) may impact their niche differentiation and geographic limits.

## Materials and Methods

### Study species and ecology: evolutionary history and current ecology

**Figure 2:**
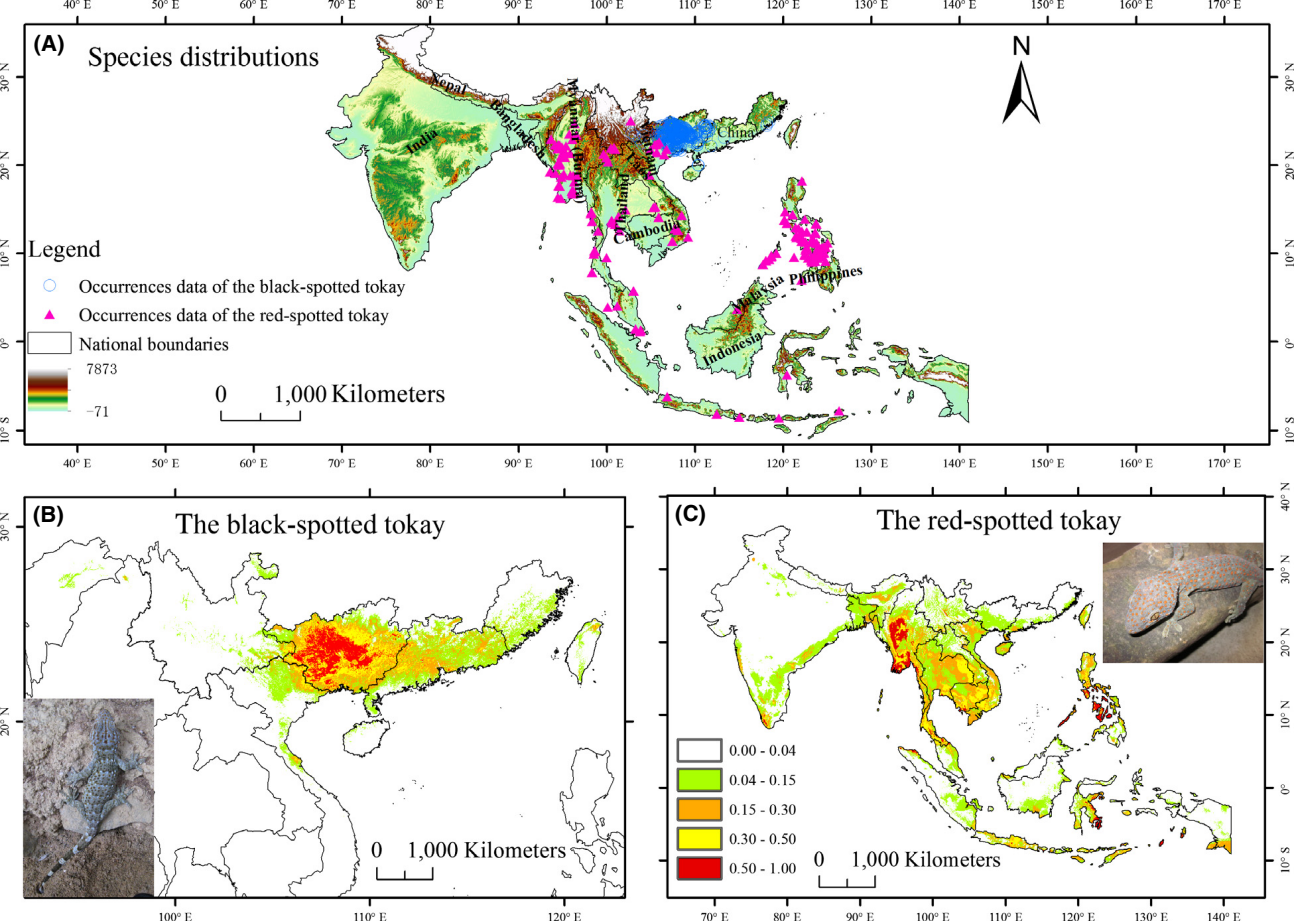
(A) Species sample localities for the black-spotted tokay and the red-spotted tokay, respectively. Blue circles represent the black-spotted tokay, while pink triangles represent the red-spotted tokay. (B) and (C) predicted potential niches of the black-spotted tokay and the red-spotted tokay as generated in MaxEnt, respectively.

The evolutionary history of *Gekko gecko* remains poorly understood. The divergence time of *Gekko gecko* was estimated at 65 million years ago (Gamble et al. 2010), which is sufficiently long for ecological niche divergence. The “black” form is currently known from southern China (provinces of Fujian, Guangdong, Guangxi, and Yunnan) and northern Vietnam (southwards to Quang Binh Province) (Rösler et al. 2011), while the “red” form has a much wider distribution, ranging from northeast India to Nepal and Bangladesh and throughout Southeast Asia, Philippines to Indonesia and also on the Australasian Archipelago and western New Guinea (Fig.2A). Most of the “black” form inhabit rock crevices of the karst hills (Yuan and Li 2008), while the “red” form is mainly distributed in rainforest areas. The habitat use and distinct range limits of the “black” and the “red” forms differ which might have resulted from niche divergence. Nevertheless, limited qualitative investigations of niche divergence are available, which may provide further insights into taxonomic status and niche differentiation.

### Distribution and environmental data

We compiled a dataset of 2582 occurrence records covering currently recognized overall ranges for the black- and red-spotted tokay. Locality data were taken from a variety of sources including field notes, museum catalogs, and the HerpNET database (http://www.herpnet.org/). Additional information was also obtained from a variety of published sources including *Fauna Sinica Reptilia, Vol.2 Squamates Lacertilia* (Zhao et al. 1999)*, Herpetology of China* (Zhao and Adler 1993)*, Zootaxa, Zoological Journal of the Linnean Society, Bonn Zoological Bulletin, Asian Herpetological Research, Zoological Research, Acta Herpetologica Information, Acta Herpetologica Sinica, Acta Zootaxonomica Sinica*, *Sichuan Journal of Zoology,* and *Chinese Journal of Zoology*. The taxonomic accuracy and the most precise locality information of all museum specimen records were verified. To reduce potential errors from HerpNET, uncertain information of distributions and identifications, such as isolated and questionable distribution records, was checked and removed. We omitted the invasive localities where *G. gecko* were introduced into Hawaii, Florida, Texas, Belize, and several Caribbean islands. Our final checklist contains 955 records, with 556 records (unique 225 records) for the “red” form and 399 records (unique 389 records) for the “black” form (Fig.2A). When available, we used geographical coordinates from museum databases or literature; otherwise, we used Google Earth to estimate coordinates from their locality description.

A total of 21 environmental variables with a spatial resolution of 30 arc sec (approximately 1 km²) were used to construct the ecological niche model. We used 19 bioclimatic variables available from the WorldClim database (Hijmans et al. 2005, http://www.worldclim.org). The variables include estimates of annual means, seasonal extremes, and seasonal variation in temperature and precipitation. In addition, we used the mean Normalized Difference Vegetation Index (NDVI), representing an estimate of the density or sparseness of vegetation in each grid cell and therefore a proxy for biotic competitive environment (Nakazato et al. 2010). NDVI was obtained from Advanced Very High-Resolution Radiometer (AVHRR) record of the average 16-day changes in the photosynthetic activity of terrestrial vegetation. NDVI is available at https://lpdaac.usgs.gov/get_data maintained by the NASA Land Processes Distributed Active Archive Center (LP DAAC), USGS/Earth Resources Observation and Science (EROS) Center, Sioux Falls, South Dakota. Finally, elevation data, which were derived from GTOPO30 (i.e., a global digital elevation model with a horizontal grid spacing of 30 arc sec), were used to represent habitat heterogeneity (Kerr et al. 2001; Huang et al. 2011) and are available at http://eros.usgs.gov/. All variables are likely to be important for species distributions based on prior studies (e.g., Currie 1991; Kerr et al. 2001; Hawkins et al. 2003; Costa et al. 2007; Huang et al. 2011).

### Ecological niche modeling

We used the maximum entropy algorithm implemented in MaxEnt v3.3 (Phillips et al. 2006) to predict each species potential distributions from the occurrence records. The MaxEnt model generally performs better than other ecological niche modeling, and has been utilized extensively as it was available (Elith et al. 2006; Phillips et al. 2006). The MaxEnt model works by evaluating the environmental suitability of each grid cell in the study area as a function of environmental variables at that cell, and calculates the most important environmental predictors for the niche of each species.

To build models, we removed highly correlative (*R* > 0.95) (Wellenreuther et al. 2012) variables prior to analysis. 75% occurrence data were used for model training and 25% for model testing to evaluate the accuracy of each model. We evaluated the model fit by calculating the area under the curve (AUC) of the receiver operating characteristic (ROC) plot (Phillips et al. 2006). The AUC value ranges from 0 to 1: an AUC < 0.5 indicates poor performing, an AUC 0.7-0.9 indicates a very good fit, and an AUC > 0.90 is considered excellent (Swets 1988; Elith 2002). We used default values for the convergence threshold (10^−5^), 100 replicate bootstrap samples and 5000 iterations to give adequate sampling for convergence. To evaluate which variables were the most important in the model, we ran a jackknife test. The variable with the highest gain contributes the most useful information to the model, whereas the variable that decreases the gain the most contains the most information that is not found in other variables. Finally, for each species, we analyzed (1) the occurrence data regress against latitude and longitude to investigate their distribution trends and (2) the environmental suitability index generated by the niche model regress against latitude and longitude to evaluate their effects on niche differences (Wellenreuther et al. 2012).

### Tests for ecological niche divergence

#### Niche identity test

This procedure is used to test whether the habitat suitability scores of the “red” form and the “black” form generated by the ecological niche modeling (ENM) have significant ecological differences (Warren et al. 2008, 2010). We calculated the niche identity test in ENMTools following the methods described in Warren et al. (2008). Schoener's *D* (Warren et al. 2008) and Hellinger's-based *I* (Schoener 1968) were used to measure niche identity, which was calculated by comparing the estimates of habitat suitability from the ENM. Schoener's *D* assumes that the suitability scores are proportional to species abundance, whereas Hellinger's-based *I* measures the probability distributions of two ecological niche models (Warren et al. 2010). Both similarity metrics range from 0 (no niche overlap) to 1 (identical niches). Bootstrapping with 100 replicates was used to calculate a null distribution.

#### Background test

We performed background tests to evaluate whether the potential ecological niches of the “red” form and the “black” form are more different from one another than expected based on the differences in the environment they occurred (Warren et al. 2008). For example, the niche model for the focal species (e.g., the “red” form) is compared to a series of pseudoreplicate models generated by randomly sampling the “background” of its sister form (e.g., the “black” form; Warren et al. 2008;). *G*. gecko has been recognized as a sit-and-wait forager (e.g., Aowphol et al. 2006), and its dispersal distance is poorly understood. Therefore, we selected the background by creating a minimum convex hull polygon using ArcMap and Hawth's Tools and generated 10000 random points from background. The test was carried out in both directions (randomization of the “black” and the “red” forms occurrences). To conduct each background test, we performed 100 pseudoreplicates for each species pair tested. Schoener's *D* and Hellinger's-based *I*, which calculated from the observed niche overlap, were compared to a null distribution of 100 replicates pseudoreplicate models overlap values (Warren et al. 2008).

### Point-based analysis

To assess whether ecological niche space of the “red” form and the “black” form was differentiated, we performed a point-based analysis. We extracted pixel values for each of the 21 environmental variables at each point site. A principal components analysis (PCA) was used to convert the original 21 climatic variables to principal components that explained most of the variability. A multivariate analysis of variance (MANOVA) was then conducted to examine significant difference between ecological niches of the “black” form and the “red” form. The taxon was the fixed factor, and PCA scores were the independent variables. All statistical tests were conducted with SPSS 19.0 (IBM Corp. 2010).

## Results

### Ecological niches

**Figure 3:**
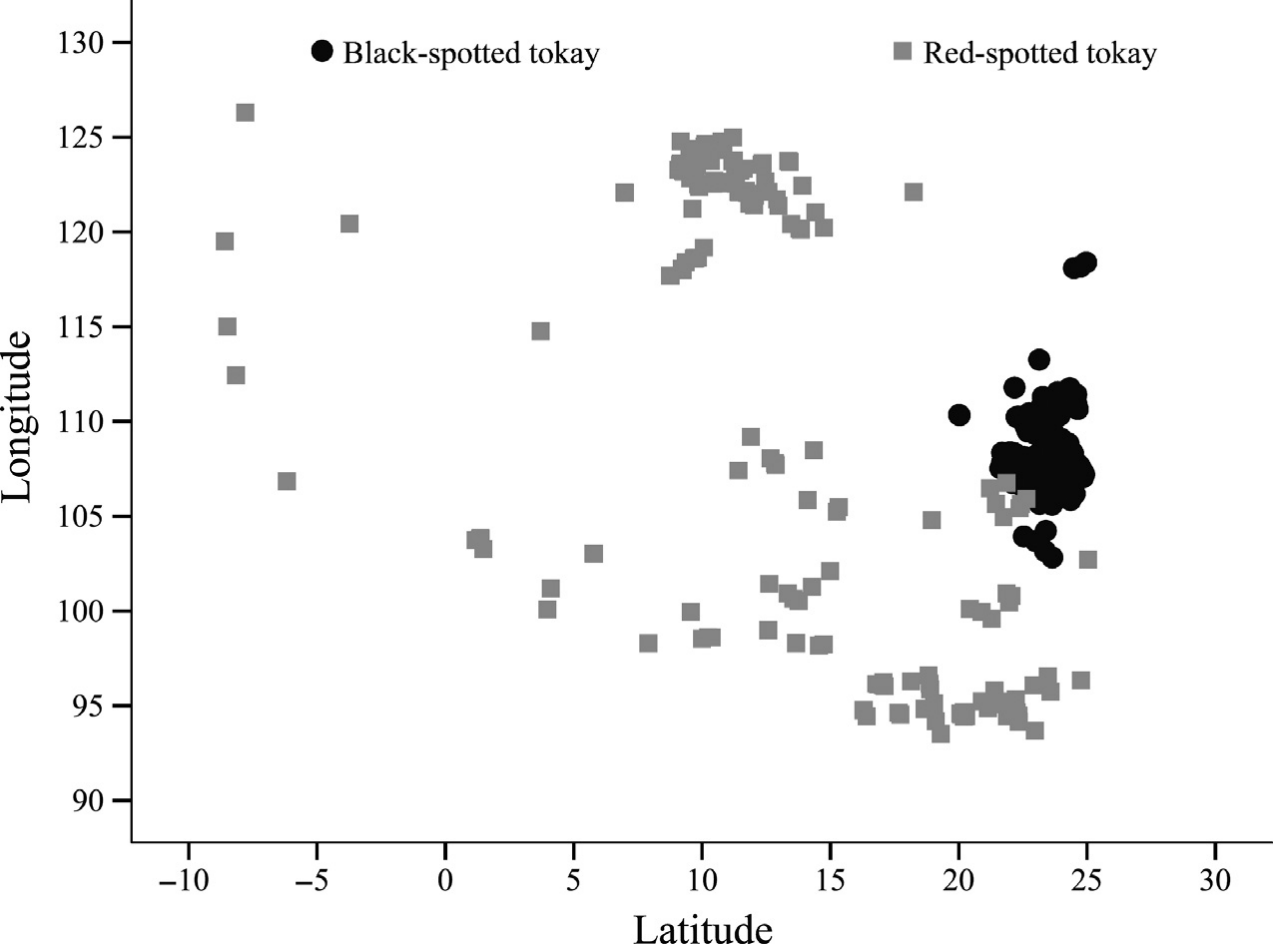
Species occurrence records with respect to latitude against longitude.

Visualizing the occurrence data showed that the “red” form has larger distributions than the “black” form (Fig.2A). The overall geographic range for the “black” form ranges from 102.83 to 118.39° longitude and from 20.00 to 24.96° latitude, while the range for the “red” form spanned from 93.53 to 126.30° longitude and from −8.6 to 25.04° latitude (Fig.3).

The predicted distribution of the “black” form and the “red” form is shown in Fig.2B,C. The potential distribution of the “red” form is almost three times as large as that of the “black” form (prevalence of 0.09 and 0.03, respectively). The area under the curve (AUC) values were 0.982, SD = ± 0.002 and 0.966 ± 0.02, respectively, for the “red” form and the “black” form, indicating robust model performance. The predicted distribution for the “black” form yielded a projected distribution which was mostly restricted to southeastern edge of the Yunnan–Guizhou Plateau and surrounding mountains, whereas the predicted distribution for the “red” form showed a little overprediction. Compared with the “black” form, the “red” form exhibited relatively low unique sample sites, although the AUC values indicated that meaningful models had been produced, such as potential distribution (e.g., southern India) in the relatively poor sampling regions (Fig.2C).

**Figure 4:**
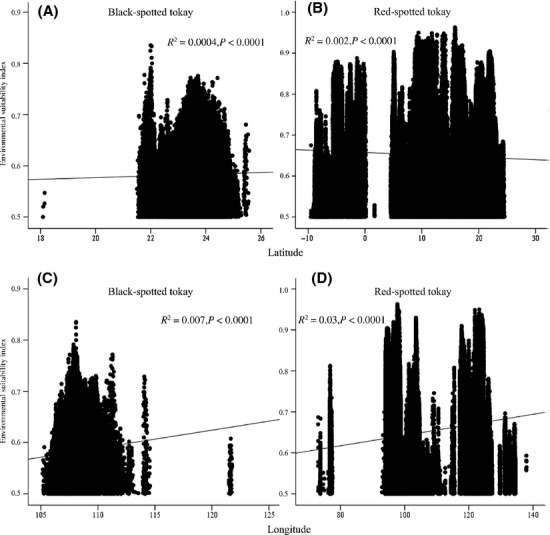
Environmental suitability index (logistic probability) against latitude: (A) the black-spotted tokay (*R*^2^ = 0.0004, *P* < 0.0001), (B) the red-spotted tokay (*R*^2^ = 0.002, *P* < 0.0001), and longitude: (C) the black-spotted tokay (*R*^2^ = 0.007, *P* < 0.0001) and (D) the red-spotted tokay (*R*^2^ = 0.03, *P* < 0.0001).

The environmental suitability index was extracted from the average predicted distribution using minimum training presence. We excluded environmental suitability indexes lower than 0.5 for computational efficiency. Regressions of environmental suitability index versus latitude were highly significant (Fig.4) but with low predictive power (Fig.4) by a low *R*-value (“black” form: *R*^2^ = 0.0004, *P* < 0.0001, intercept = 0.54; “red” form: *R*^2^ = 0.002, *P* < 0.0001, intercept = 0.66). The results of the environmental suitability index regressed against longitude were similar to these of latitude (“black” form: *R*^2^ = 0.007, intercept = 0.20, *P* < 0.0001; “red” form: *R*^2^ = 0.03, intercept = 0.51, *P* < 0.0001). There was no longitudinal overlap for the “black” and the “red” forms (Fig.4).

MaxEnt analysis revealed that “Isothermality” (48.4% of contributions) was the highest ranked variable for the “black” form, while “Temperature Seasonality” (14.8% of contributions) was the highest for the “red” form (Table1). Both variables showed the highest gain when used in isolation for models (Table1). But the explaining amount of variation for the “red” form, “Precipitation of Warmest Quarter” (13.6% of contributions) and “Temperature Annual Range” (11.3% of contributions), may be equally important to “Temperature Seasonality.” NDVI decreased the most for the “black” form, while “Precipitation of Warmest Quarter” for the “red” form.

**Table 1:** The estimate results of relative contributions of the environmental variables to the MaxEnt model for the black-spotted tokay and the red-spotted tokay, respectively. Isothermality is calculated as (mean of monthly (maximum Temperature - minimum Temperature))/(Temperature Annual Range) ×100). The environmental variable with highest gain having the most useful information by itself to the model. The environmental variable that decreases the gain the most having the most information that is not present in the other variables.

Environmental layer	% Contribution for the “black” form	% Contribution for the “red” form
Isothermality	48.4	3.2
Mean temperature of coldest quarter	11.2	1.8
Mean diurnal range	9.7	1.5
Precipitation of warmest quarter	5.6	13.6
Mean temperature of wettest quarter	5.3	1.5
NDVI	5.1	2.4
Precipitation of wettest month	3.7	4.3
Temperature annual range	2.3	11.3
Precipitation seasonality	2	8.8
Mean temperature of driest quarter	1.8	3.7
Min temperature of coldest month	1.6	4.7
Temperature seasonality	1.3	14.8
Elevation	0.9	2.5
Annual mean temperature	0.4	1.7
Precipitation of driest month	0.4	9.6
Precipitation of driest quarter	0.2	3.6
Max temperature of warmest month	0.1	0.4
Mean temperature of warmest quarter	0	1.7
Precipitation of wettest quarter	0	1.3
Precipitation of coldest quarter	0	7.6
Annual precipitation	0	0.1

### Sister species comparisons: niche similarity tests

**Figure 5:**
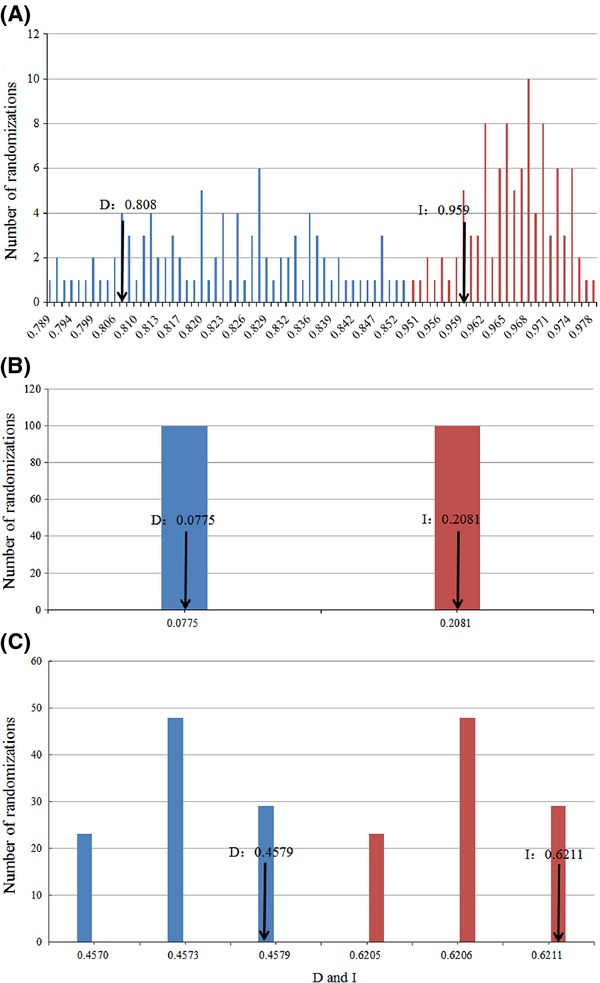
Niche-overlap values (arrows) for Schoener's *D* and Hellinger's-based *I* are compared with a null distribution. (A) Niche identity tests; (B) Background test with the black-spotted tokay as focal species, the ranges of red-spotted tokay as background; (C) Background test with the red-spotted tokay as focal species, the ranges of black-spotted tokay as background. Blue columns indicate the null distribution of *D*, while red columns indicate the null distribution of *I*. The *X*-axis indicates the value of *D* and *I*, whereas the *Y*-axis shows the number of randomizations. The arrow indicates the value in actual MaxEnt runs.

Based on the niche identity test, the observed values of *D* and *I* measuring the current niche overlap between the “red” form and the “black” form were significantly different (*t*-test, df = 99, *P* < 0.001) from the null distributions (Schoener's *D* = 0.81, Hellinger's-based *I* = 0.96, Fig.5A). For the background test, the results indicated nonsignificant divergence between the focal potential niches of the “black” form and the “red” form when taken into account the random background points for the “red” form (*t*-test, df = 99, *P* > 0.05 for Schoener's *D*; *t*-test, df = 99, *P* > 0.05 for Hellinger's-based *I;* Fig.5B). When the random background points for the “black” are considered, a similar divergence was found (*t*-test, df = 99, *P* < 0.001 for Schoener's *D*; *t*-test, df = 99, *P* < 0.001 for Hellinger's-based *I*; Fig.5C).

### Point-based analysis

**Figure 6:**
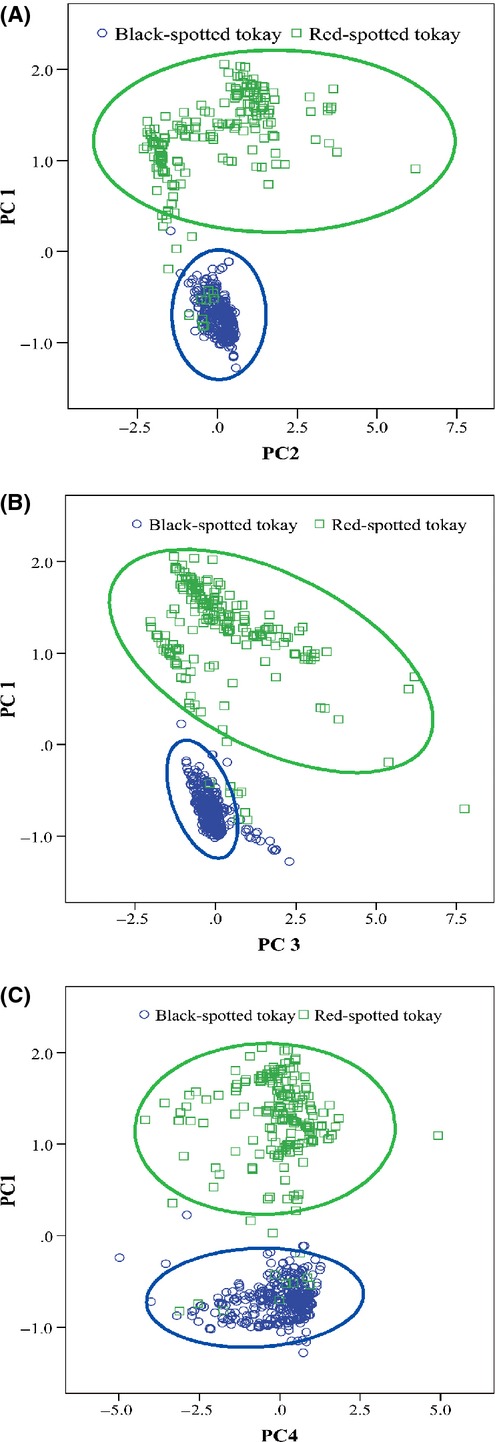
Principal components analysis of niche differences between the black-spotted tokay and the red-spotted tokay (framed by circle and square, respectively). (A) *X*-axis indicates PC2, and *Y*-axis indicates PC1. (B) *X*-axis indicates PC3, and *Y*-axis indicates PC1. (C) *X*-axis indicates PC4, and *Y*-axis indicates PC1.

The first four principal components (PC) from the PCA cumulatively explain more than the 85% of the variation, with each PC responsible for 40, 20, 14, and 11% of the variation, respectively. PC1 was interpreted as explaining variation from temperature variables (Min Temperature of Coldest Month, Mean Temperature of Coldest Quarter, Temperature Seasonality, Mean Temperature of Driest Quarter, Isothermality, and Temperature Annual Range) (Table2). PC2 was correlated with variables describing Precipitation Seasonality, Max Temperature of Warmest Month, Precipitation of Driest Quarter, and Precipitation of Driest Month (Table2). PC3 loaded mainly on Precipitation of Wettest Quarter as well as Precipitation of Wettest Month, while PC4 dominated by Precipitation of Wettest Month (Table2). The MANOVA analyses showed that environmental conditions differed significantly between the two forms (Pillai's trace = 103.87, *P* < 0.001; Fig.6).

**Table 2:** Results of the principal components analysis of 21 environmental variables. Max denotes the maximum value and min denotes the minimum value.

	Name of environmental data	PC1	PC2	PC3	PC4
bio1	Annual mean temperature	0.892	0.365	−0.225	0.083
bio2	Mean diurnal range: mean of monthly (max temp–min temp)	0.080	0.607	−0.202	−0.488
bio3	Isothermality: (bio2/bio7) × 100	0.941	−0.137	−0.041	−0.255
bio4	Temperature seasonality (standard deviation × 100)	−0.953	−0.097	−0.050	0.253
bio5	Max temperature of warmest month	0.198	0.786	−0.430	0.249
bio6	Min temperature of coldest month	0.980	0.064	−0.065	−0.022
bio7	Temperature annual range (P5–P6)	−0.927	0.220	−0.089	0.113
bio8	Mean temperature of wettest quarter	−0.143	0.270	−0.421	0.704
bio9	Mean temperature of driest quarter	0.952	0.239	−0.117	−0.056
bio10	Mean temperature of warmest quarter	0.089	0.540	−0.501	0.626
bio11	Mean temperature of coldest quarter	0.962	0.225	−0.076	−0.092
bio12	Annual precipitation	0.659	0.012	0.643	0.333
bio13	Precipitation of wettest month	0.433	0.334	0.765	0.303
bio14	Precipitation of driest month	0.367	−0.781	−0.142	0.311
bio15	Precipitation seasonality (coefficient of variation)	−0.320	0.798	0.437	−0.067
bio16	Precipitation of wettest quarter	0.388	0.341	0.783	0.313
bio17	Precipitation of driest quarter	0.429	−0.786	−0.138	0.281
bio18	Precipitation of warmest quarter	−0.559	−0.308	0.428	0.463
bio19	Precipitation of coldest quarter	0.641	−0.626	−0.086	0.115
Elevation	GTOPO30	−0.306	−0.121	0.258	−0.521
NDVI	Normalized difference vegetation index	−0.235	0.081	−0.040	−0.026

## Discussion

### Ecological differences between the black- and red-spotted tokay

Environmental characterization and accompanying ecological niche modeling can provide additional insight into evaluating taxonomic status and niche distinctiveness (Nakazato et al. 2010). To our knowledge, this is the first study explicitly using ecological niche models to understand ecological divergence between the black- and the red-spotted tokay. Our results showed little geographical overlap between the “black” form and the “red” form (Fig.2), indicating that each has a unique range. Both niche identity test and background test suggested the significant ecological niche divergence between the two forms, supporting the involvement of ecological differentiation in speciation. We also explored the ecological differentiation by ENM using the distinct mitochondrial DNA lineages data such as corresponding to Rösler et al. (2011) and Wang et al. (2012), and the results indicated that they are under significantly different ecological conditions (data not shown here).

Previous studies have demonstrated morphological and genetic differentiation between the “black” form and the “red” form (Zhang et al. 1997; Qing et al. 2007; Qin et al. 2009; Wang et al. 2012). Although our analyses did not test whether ecological niche shifts directly lead to speciation events, it is clear that ecology is closely related to persistent species differentiation (Nakazato et al. 2010). Divergent natural selection through abiotic pressures can cause adaptive divergence and ultimately speciation (Rissler and Apodaca 2007). Streelman and Danley (2003) suggested that the initial axis of divergence occurs in many adaptive radiations, that is, followed by divergence in morphology and communication during the vertebrate evolutionary radiation. Based on the substantial ecological differentiation detected, we argue that ecological niches are generally not conserved among the tokay gecko species. Our results contribute to additional perspective that the “black” form and the “red” form are differentiated based on significant ecological differentiation and one or more climatic variables.

### Abiotic factors shaping the black- and red-spotted tokay niche differentiation

Adaptation to local environmental conditions is a leading force in shaping morphological evolution and speciation (Schluter 2000, 2001; Nakazato et al. 2010). Our ecological niche modeling predicted potential distributions with high confidence, indicating that their geographic ranges are substantially influenced by the abiotic environmental factors on which we focused. Climatic variables in general are more important predictors of species distribution than NDVI and altitude (Table1). Environmental variables contributed differently to each species, mainly “Isothermality” for the “black” form, and “Temperature Seasonality,” “Precipitation of Warmest Quarter,” and “Temperature Annual Range” together for the “red” form (Table1). These were demonstrated separately by PCA analysis in ecological space (Fig. 6). If species adapt to different environments, then these environmental variables should limit their distributions to a certain extent (Nakazato et al. 2010). Thus, the sensitivity to climatic transitions suggested that phenotypic divergence might have been driven by shifts into new ecological niches created by climate. It is likely that adaptation of each species to different local conditions has led to niche differentiation and was accomplished by acquisition of unique morphological or physiological innovations (Nakazato et al. 2010), namely resulting in the morphological replacement of two morphs. In addition, the adaptation may reflect differential abilities to survive and reproduce in varying local environmental conditions, for example, competitive water and nutrient resources in a particular zone (Khimoun et al. 2012).

Substantial ecological differentiation between the “black” form and the “red” form pair was detected by significant niche similarity tests. The niche identity test based on the ENM also suggested they had highly significantly different niches (Fig.4A). The background test gave counter-intuitive result for the pair, which they were more similar to the background from the range of the “red” form than expected by chance (Fig.4B), while they were less similar from the range of “black” form than expected by chance (Fig.4C). Although we chose the minimum convex polygon of known occurrences as “background,” we also performed the sensitivity analysis to conduct background tests using a priori definition of “background” with countries ranges where we know their distributions at the current time (Fig.2A), with results the same as MCP (data not shown here). These results indicated that it was possible that the heterogeneity of the environmental background led to significantly similarity in one direction, but significantly less similarity in the other (Nakazato et al. 2010). In other words, the environmental background of the “red” form was possibly more heterogeneous, while occurrences of the “black” form look more like known locations of the “red” form than the heterogeneous background. Therefore, data from niche similarity tests suggested niche conservatism for the “black” form and the “red” form used a subset of the “black” form habitat.

### Applications of environmental characterization and ENM

Ecological niche models based on environmental factors are a powerful tool for predicting the geographic range of species. Our results suggest that both the “black” form and the “red” form occupied a part of the geographic range where environmental conditions were suitable (Figs.2 and 3). Our results also suggest that the geographic range of the “red” form should be larger than the predicted range of the “black” form (Fig.2). The potential distribution results for them may guide us to perform more detailed field surveys in the future, likely accelerating the discovery of new distribution, and effectively identify in current ranges for conservation (Raxworthy et al. 2003; Huang et al. 2011), particularly for the “black” form, which is listed as a “Class II State Key Protected Animal” in China. The tokay gecko is quickly becoming a threatened species in Philippines and Vietnam due to indiscriminate hunting, trading for medicinal purposes, increasing urbanization and habitat lost. If it is necessary to establish conservation priority for the tokay gecko, these available data will be highly informative to conservation efforts. In addition, invasive species are an issue of great concern globally. The tokay gecko is considered as an invasive species as they were introduced into Hawaii, Florida, Texas, Belize, and several Caribbean islands in the late 1980s and early 1990s. Our niche modeling results broadly estimate the most important environmental predictors and requirements that a species may need to identify suitable establishment locations in advance and help decision-makers and environmental protection officials take preventative measures or make rapid responses. Consequently, the ecological niche modeling usually emphasizes the role of the environmental factors, but this interpretation should be regarded cautiously, because the role of biotic factors such as dispersal limitation and biotic interactions (e.g., competition) and historical processes shaping the absence of range overlap where they may share similar environmental conditions are usually ignored and difficult to include in ecological modeling (Costa et al. 2007; Sillero 2011).

## Conclusions

To conclude, our results revealed that niche differentiation between the black- and red-spotted tokay was significantly detected. Such ecological niche divergence occurring in allopatry has often been inferred to, or at least in line with, the ecological speciation hypothesis (Schluter 2000; Rundle and Nosil 2005; Wellenreuther et al. 2012). Our results imply that it is important to quantify niche differences across geographic scales, aiding to define cryptic species. In other words, our results further supported taxonomic suggestions of Rösler et al. (2011), that is, the “black” form and “red” form were taxonomically distinct, which the “red” form is *G. g. gecko*, while the “black” form is regarded as *G. reevesii*. Our results also suggested that divergence in physiological temperature and precipitation was the most likely factors that can explain their different distributions. It could be a major driver of the range limits and niche divergence across the entire range. However, caution should be taken to infer the niche divergence, and more mechanistic and experimental studies of individuals are required in the field or in the laboratory (e.g., comprehensive geographic sampling and a functional characterization of differences in resource use; Losos 2000; Wellenreuther et al. 2012).
